# Evaluating Whether Nonimmersion Virtual Reality Simulation Training Improves Nursing Competency in Isolation Wards: Randomized Controlled Trial

**DOI:** 10.2196/63131

**Published:** 2025-01-17

**Authors:** Dandan Zhang, MuLi Fu, Jianzhong Zhang, Yuxuan Li, Li Chen, Yong-Jun Chen, Zhefeng Zhong, Yin-Ping Zhang

**Affiliations:** 1 School of Nursing, Xi'an Jiaotong University Health Science Center Xi'an China; 2 Affiliated Nanhua Hospital, Hengyang Medical School, University of South China Hengyang China; 3 The First Affiliated Hospital, Hengyang Medical School, University of South China Hengyang China; 4 School of Economy & Finance, Xi'an Jiaotong University Xi'an China

**Keywords:** virtual reality simulation, isolation ward, preparedness, pandemic, nurse

## Abstract

**Background:**

During infectious disease outbreaks such as the COVID-19 pandemic, nurses are crucial in patient care and public health safety; however, they face challenges such as inadequate training and high stress in isolation wards. Virtual reality (VR) technology offers innovative training solutions to enhance nurses’ clinical skills and preparedness. However, extensive studies on its effectiveness in isolation ward environments are still limited.

**Objective:**

This study aims to develop a nonimmersive VR (NIVR) simulation training program for isolation wards and further validate its feasibility and training effectiveness in aiding nurses in adapting to isolation ward settings.

**Methods:**

This study was a prospective, parallel, open-label, randomized controlled trial. A total of 90 nurses from 3 hospitals in China were randomly assigned to either the control or intervention group, with 45 (50%) individuals in each group. Both groups received training on isolation ward layout and nursing procedures. The control group underwent a 4-hour conventional training session consisting of 2 hours of face-to-face lectures and 2 hours of ward visits. The intervention group received a 4-hour NIVR simulation training session. Subsequently, both groups completed approximately 4 hours of emergency drills and assessments.

**Results:**

After the intervention, there were no significant differences in theoretical test or performance assessment scores between the 2 groups (*t*_88_=–0.30, *P*=.75; Cohen *d*=–0.06; *z* score=0.00, *P*>.99), using a 2-tailed *t* test. However, the intervention group completed 6 tasks faster than the control group (*t*_88_=5.10, *P*<.001; Cohen *d*=1.08), with an average reduction of about 3 minutes (control group: mean 43.91, SD 2.99 min; intervention group: mean 40.77, SD 2.85 min). Notably, they completed task 3 (patient reception inward) and task 6 (exiting the isolation area) significantly quicker (*t*_88_=3.22, *P*=.002; Cohen *d*=0.68; *t*_88_=3.03, *P*=.003; Cohen *d*=0.64*,* respectively), with no significant differences for the other tasks.

**Conclusions:**

This study highlights the potential of NIVR simulation training for nurses working in isolation wards. Although NIVR simulation training does not significantly surpass traditional methods in imparting theoretical knowledge, it does reduce task completion time for specific activities. Its capacity for safe, repetitive practice and realistic scenario simulation makes NIVR a valuable tool in medical education. Further research and optimization of VR simulation training programs are recommended to enhance nurses’ practical skills and pandemic preparedness.

**Trial Registration:**

Chinese Clinical Trial Registry ChiCTR240083155; https://www.chictr.org.cn/hvshowproject.html?id=250356&v=1.0

## Introduction

### Background

The world faced the challenge of outbreaks of new infectious diseases. During the COVID-19 pandemic, nurses played a vital role in ensuring that patients received personalized, high-quality care while also being essential in patient management, infection prevention, and public health safety [1]. However, there were shortcomings in nurses’ abilities to respond to the COVID-19 pandemic [2-6]. Therefore, it was necessary for nurses to receive adequate guidance and training to ensure a safe work environment, protect themselves, and effectively manage the pandemic [1]. To address this issue, the *National Nursing Career Development Plan (2021-2025)* released by the National Health Commission of China explicitly proposed targeted training in scarce nursing specialties, such as infectious diseases [7]. With national policy support, training programs for infectious disease specialist nurses are gradually developing. However, existing programs still face challenges, such as insufficient training, incomplete content, unitary training patterns, limited scenario simulations or emergency drills, and insufficient knowledge of ethics and law, particularly in professional training programs for nurses in the COVID-19 pandemic response [2-6,8-12].

When an pandemic breaks out, isolation wards protect health care workers and patients from infection. Studies have shown that the mental stress and occupational safety of frontline health care workers were linked to unfamiliarity with isolation wards [13,14]. Therefore, it is crucial to train nurses to familiarize them with isolation wards before they enter the quarantine area. Building on the COVID-19 pandemic response experience, health care institutions have intensified emergency training programs for professionals [15]. Nurses need to understand the spatial layout and division of the functional regions in isolation wards and critical aspects, such as standard procedures, personnel management, and infection prevention and control. The training and support aimed to help nurses acclimate to the work environment before entering the isolation area, thus reducing stress and anxiety stemming from unfamiliar surroundings, enhancing preparedness and teamwork abilities, and contributing to a more efficient response. However, studies on related topics remain relatively scarce.

Virtual reality (VR) technology uses digital techniques to create 3D VR environments, allowing users to interact with these environments through head-mounted displays, body sensors, and direct input devices [16]. On the basis of the level of immersion, VR is categorized into immersive VR, semi-immersive VR, and nonimmersive VR (NIVR) [17]. NIVR presents a virtual environment via a computer screen or mobile device, allowing users to interact using a keyboard, mouse, or touch devices [18]. Although NIVR offers a relatively diminished sense of immersion, its low cost, ease of deployment, and lack of a requirement for special equipment confer significant application potential across various scenarios, particularly in training [19-21]. Users can easily access these scenarios on PCs or mobile devices, overcoming temporal and spatial limitations.

Studies indicate that in nursing education and clinical practice, the effectiveness of NIVR can be comparable to, or even surpass, traditional simulation training [22-24]. This technology can effectively simulate complex medical procedures and nursing scenarios, allowing trainees to practice repeatedly in a safe, controlled environment, thus enhancing clinical skills, decision-making ability, and critical thinking [22,25-33]. In addition, a study indicates that the design characteristics of VR simulations encompass patient management content, the implementation of various scenarios and NIVR experiences lasting >30 minutes, and the provision of feedback following the conclusion of the scenarios. These strategies can significantly enhance the effectiveness of VR simulations [34]. During the COVID-19 pandemic, VR proved helpful in addressing the training needs of medical students; however, the lack of hands-on practice might impact the development of clinical skills [35,36]. Therefore, it is necessary to explore integrated training that combines VR with hands-on practice. Yang et al [37] demonstrated that combining NIVR and face-to-face simulation training effectively enhances nursing clinical judgment and encourages student progress in comprehensive simulations. Thus, when integrating VR with traditional training methods, it is crucial to understand the strengths and limitations of each technology and adopt the best practices [29,30,38]. Studies should explore how to integrate these technologies more effectively into the training process, driving innovation and progress in medical education.

The COVID-19 pandemic has underscored the necessity of advancing VR-based remote education [33]. NIVR has advantages in remote virtual training and is increasingly being adopted in nursing degree programs and preservice education [13,33,39]. During outbreaks, practical clinical applications of NIVR simulation training cover multiple aspects, such as infection prevention and control, the use of personal protective equipment [40-42]. It also includes training for managing patients in critical condition, such as basic life support, advanced cardiac life support, and extracorporeal membrane oxygenation [43-47]. NIVR training related to simulating clinical scenarios and integrating clinical cases continues to evolve, with management programs for patients with COVID-19 and Ebola virus diseases among the initiatives [42,48-50]. Currently, research on using VR simulations for nurses to familiarize themselves with and adapt to isolation ward environments remains very limited.

### Objectives

The primary objective of this study was to develop an NIVR simulation training program specifically designed for isolation wards and to validate its feasibility and effectiveness in assisting nurses in adapting to this unique environment.

## Methods

### Participants

This study recruited qualified nurses from 3 comprehensive hospitals in Hengyang City, Hunan Province, from April 1 to April 20, 2024. This study included registered nurses aged 18 to 40 years, with ≥1 year of clinical experience, no previous work experience in isolation wards, good physical health, and a willingness to fully participate in the training. Nonvoluntary participants and those who withdrew midway were excluded. Before commencing the training, each participating nurse was provided with an informed consent form outlining the project training process and duration. The study was initiated after the nurses signed the consent form voluntarily.

A total of 90 eligible nurses participated in this study and were randomly divided into control and intervention groups, with 45 (50%) nurses in each group.

### Study Design

This prospective, parallel, randomized controlled trial was registered in April 2024 with the Chinese Clinical Trial Registry (registration number: ChiCTR240083155).

### Ethical Considerations

This study was approved by the ethics committee of Affiliated Nanhua Hospital, University of South China (2024-ky-044). This study complies with the Declaration of Helsinki. All participants provided written informed consent and were made aware of their right to opt out of the study at any time. All collected data are anonymous and confidential, and only the authors can access them. Upon completing the training and assessment for this study, participants will be awarded a gift worth approximately US $30 as compensation.

### Randomization and Blinding

Participant randomization into intervention and control groups was conducted using SPSS software (version 25.0; IBM Corp) to generate random numbers for a 1:1 allocation. To ensure the integrity of the allocation sequence, it was concealed using sequentially numbered, opaque, and sealed envelopes, which were only opened at the time of intervention assignment after all participants had completed their baseline assessments.

Due to the open-label nature of the study, it was not possible to blind participants or research assistants involved in the intervention. However, we implemented several measures to minimize subjective biases and ensure objective data collection. First, we scheduled intervention sessions for the 2 groups at different times to prevent cross-group communication. Second, distinct teams managed different stages of the study, ensuring that data collectors were not aware of participants’ group allocations. Moreover, outcome adjudicators remained blinded to group assignments throughout the process.

### Interventions

The control group completed the training on May 25, 2024, while the intervention group finished the training on May 26, 2024. Both groups underwent a 4-hour training session on isolation ward layout and nursing workflow in the morning. The intervention group received NIVR simulation training, while the control group received training in the form of conventional lectures and on-site ward visits. After the training, emergency drills and assessments were completed in the afternoon. The specific details of the training program are outlined subsequently.

The control group received conventional training. Participants in the control group initially received 2 hours of theoretical training. An experienced senior nurse who has worked in isolation wards for >5 years and has responded to pandemics multiple times was the lead instructor and used PowerPoint (Microsoft Corporation) presentations, images, videos, and other tools for face-to-face lectures. The topics covered in the training included an overall layout diagram of the isolation ward; the unique environment of the ward; specific distributions of clean areas, semicontaminated areas, contaminated areas, buffer zones, health care provider passages, and patient pathways; the correct personal protective measures; and disinfection. It also covered the work responsibilities and routes to the wards for the nursing shift, including procedures for entering and exiting the ward, patient admission and discharge management, methods for transferring items through pass windows, and transporting collected specimens.

Subsequently, the participants spent 2 hours familiarizing themselves with the isolation ward environment. The infectious disease building at the training site comprises 3 floors of negative pressure isolation wards. Each floor features an identical ward layout and is supervised by 2 senior isolation ward nurses as mentors. The group of 45 participants was further divided into 3 groups, each of 15 participants, with 1 group assigned to tour the first-floor ward. In the first hour, participants toured the layout of the ward under the guidance of a mentor. The following hour was dedicated to individuals or teams independently familiarizing themselves with and exploring the ward’s layout. Mentors were available on-site to address any inquiries from the participants.

**Figure 1:**
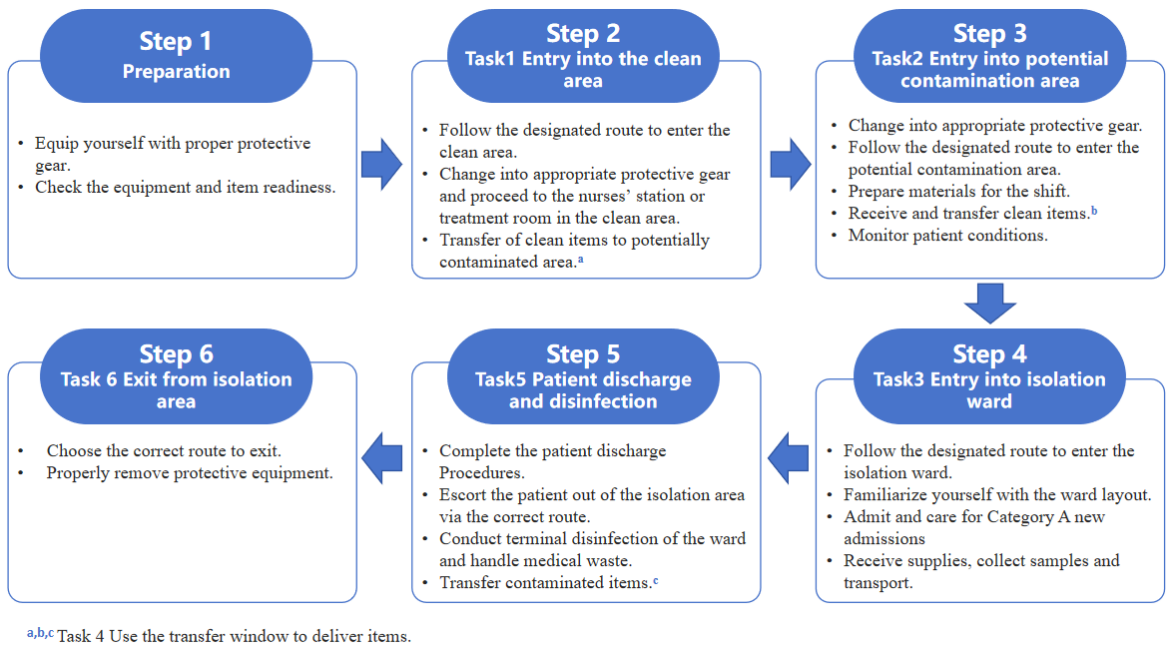
Flow diagram of the drills.

Next, 4-hour simulation drills and assessments were conducted using a team-based approach in the isolation ward to replicate the management of cases involving class A respiratory infectious diseases. The procedure for the simulation drills is provided in Figure 1. The participants were divided into 3 groups, each consisting of 15 individuals. The group leader assumed the role of the head nurse, while the members performed duties as ward nurses. The team leader scheduled shifts for 15 staff in the unit, assigning each person different shift responsibilities to simulate the management of patients in the isolation ward. The first hour was dedicated to practicing to familiarize oneself with various tasks, followed by a 3-hour Objective Structured Clinical Examination (OSCE) assessment.

The intervention group received VR simulation training. First, from February to April 2024, our study team of medical experts collaborated closely with a virtual technology team to design and develop a VR project titled “Isolation Ward Layout and Nursing Procedures.” The medical experts provided expertise on the layout and procedures of isolation wards, while the technology team used advanced VR technology to transform this guidance into a highly interactive and realistic simulation environment.

In terms of hardware, the project used a NIVR solution that presented a virtual environment via standard PCs or mobile devices, eliminating the necessity for specialized headsets. Participants could engage with the virtual environment on any compatible device using a keyboard, mouse, or touch screen. This approach enhanced accessibility and convenience, rendering NIVR training suitable for large-scale training applications, particularly in contexts where logistical simplicity and widespread access were essential.

For software development, Unreal Engine 5 (Epic Games) was used as the platform. With its powerful graphics rendering capabilities and realistic physics engine, Unreal Engine 5 enabled high-fidelity simulation of the isolation ward environment and layout. Its flexibility and scalability supported complex interactive mechanisms, providing users with an immersive learning and training experience.

**Figure 2:**
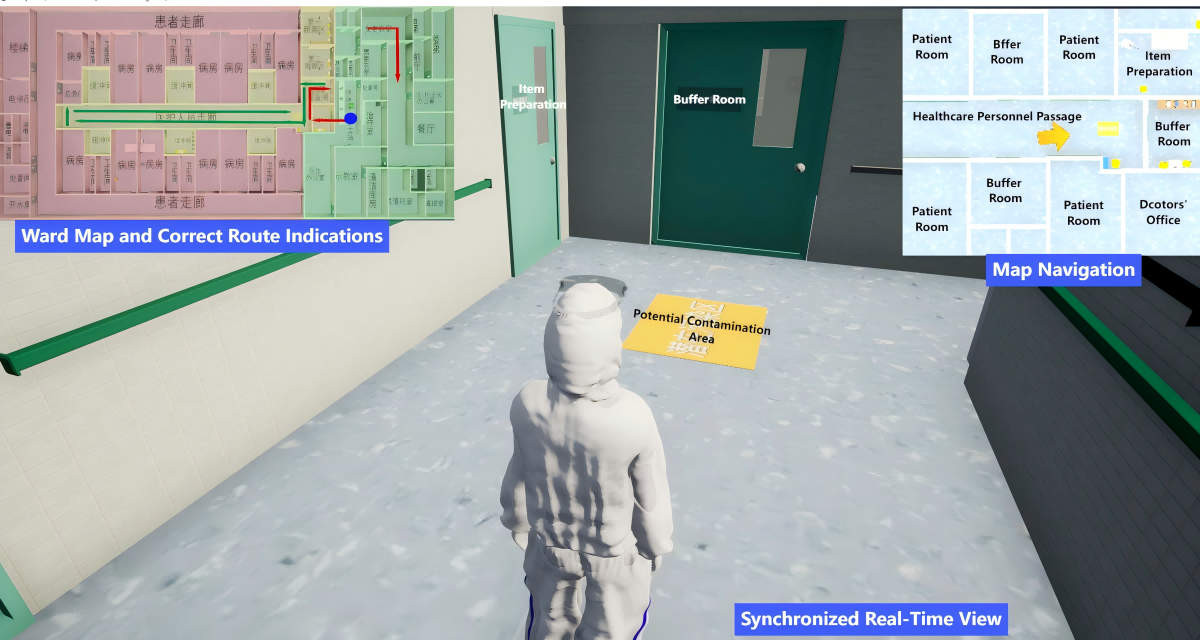
Example of 2D map navigation and 3D layout of isolation ward. The nurse’s position is indicated in real-time by a yellow arrow on a 2D navigation map, while a 3D real-life simulation of the current location is provided to help participants become familiar with the ward.

This project used a combination of 2D map navigation and interactive 3D dynamic scenes of the isolation ward (Figure 2) to demonstrate specific aspects of high-security (negative pressure) isolation wards. The scenarios presented detailed descriptions of the unique working environments and layouts within these isolation areas, setting out specific tasks for nursing personnel in different shifts. The scenes covered procedures for entering isolation areas, receiving patients with class A infectious disease upon admission, monitoring patient conditions, providing treatment and care, discharging patients, and exiting the isolation ward. Key details included protection and disinfection practices in various areas, the preparation and transfer of supplies, specimen collection, and transportation. This approach aimed to familiarize trainees with distinct area layouts, working environments, and standard procedures. Following practice sessions, assessments were administered to reinforce the learned knowledge and evaluate training effectiveness. The ability to repeat the experiments addressed the challenge of limited opportunities for trainees to engage in on-site field training within infectious disease outbreak isolation areas.

Participants underwent a 4-hour VR simulation training in a computer-simulated laboratory. An instructor provided a 30-minute demonstration and explanation of the VR simulation operations before the participants practiced various simulated tasks. Participants completed virtual simulation tasks, including approximately 3 hours of practice and postpractice evaluation. During the practice, the instructor solved the participants’ problems on-site. Subsequently, the instructor led an approximately 30-minute discussion and reporting session to address practice-related queries and share experiences.

**Figure 3:**
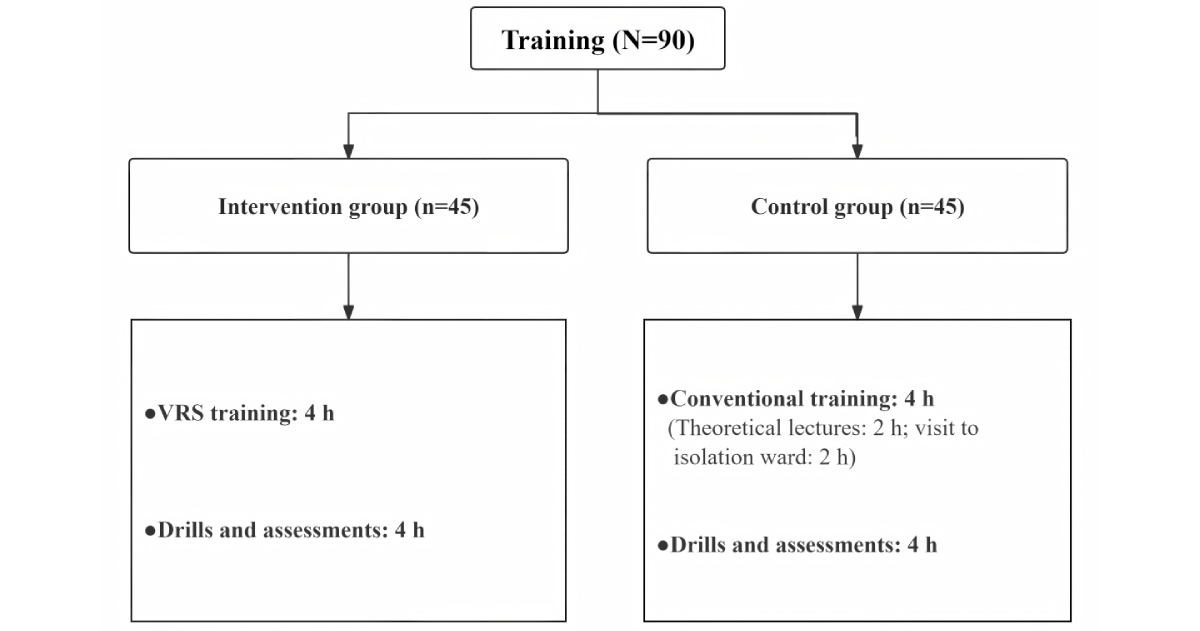
Training protocols for the 2 groups. VRS: virtual reality simulation.

For the intervention group, the content of the simulation drills and assessments lasting 4 hours was the same as that of the control group. Figure 3 illustrates the training protocols for the 2 groups.

### Measurement Indicators

#### Primary Outcome Measures

##### Theoretical Test Scores Before and After the Training

Both groups underwent web-based theoretical quizzes simultaneously before and after completing the training. The quizzes consisted of multiple-choice and fill-in-the-blank questions, with a total score of 100 points.

##### Evaluation of Drill

The assessments were conducted in the afternoon following the completion of training by the 2 groups. The 3 isolation wards were each arranged as assessment venues. Participants were randomly assigned by drawing lots, forming teams of 15 nurses each, who then entered the assessment area of a specific isolation ward. OSCE was used for the evaluation of the test, comprising 6 stations: nurse’s entry into the ward to reach the clean nurses’ station (10 points), entry into the potentially contaminated area and preparation of therapeutic and caring materials (20 points), entering the patient’s room and completing the reception of incoming patients (20 points), using the transfer window to pass items (clean item transfer window, ward transfer window, and contaminated item transfer window; 15 points), handling patient discharge and completing terminal disinfection after discharge (20 points), and nurse’s departure from the contaminated area to leave the ward (15 points), with the total score adding up to 100 points. The key scoring aspects included assessing whether the route selection aligned with the optimal path, ensuring the correct implementation of personal protective measures, appropriate use of materials, and adherence to standardized operational procedures. Each participant adhered to the team leader’s schedule, arriving at the assigned task stations, sequentially completing tasks at each station, and recording their performance at every station.

#### Secondary Outcome: Duration of Drill Task Completion

The time each group of participants spent at each station during the OSCE test was simultaneously recorded (excluding the time for wearing and removing protective equipment and performing treatment procedures).

### Statistical Analysis

Two researchers entered and verified the data and analyzed it using SPSS software (version 25.0). Continuous data were summarized with mean and SD, while categorical data were summarized using percentages. Student *t* tests (**2-tailed**) were applied to the intergroup comparisons of normally distributed continuous data. In cases of nonnormal distribution, the data were described as median (IQR), and the Mann-Whitney *U* test was used. Group comparisons for count data were conducted using *χ^2^* tests or the Fisher exact probability method. Statistical significance was denoted by 2-sided *P* values <.05.

## Results

### Demographic Data

**Figure 4:**
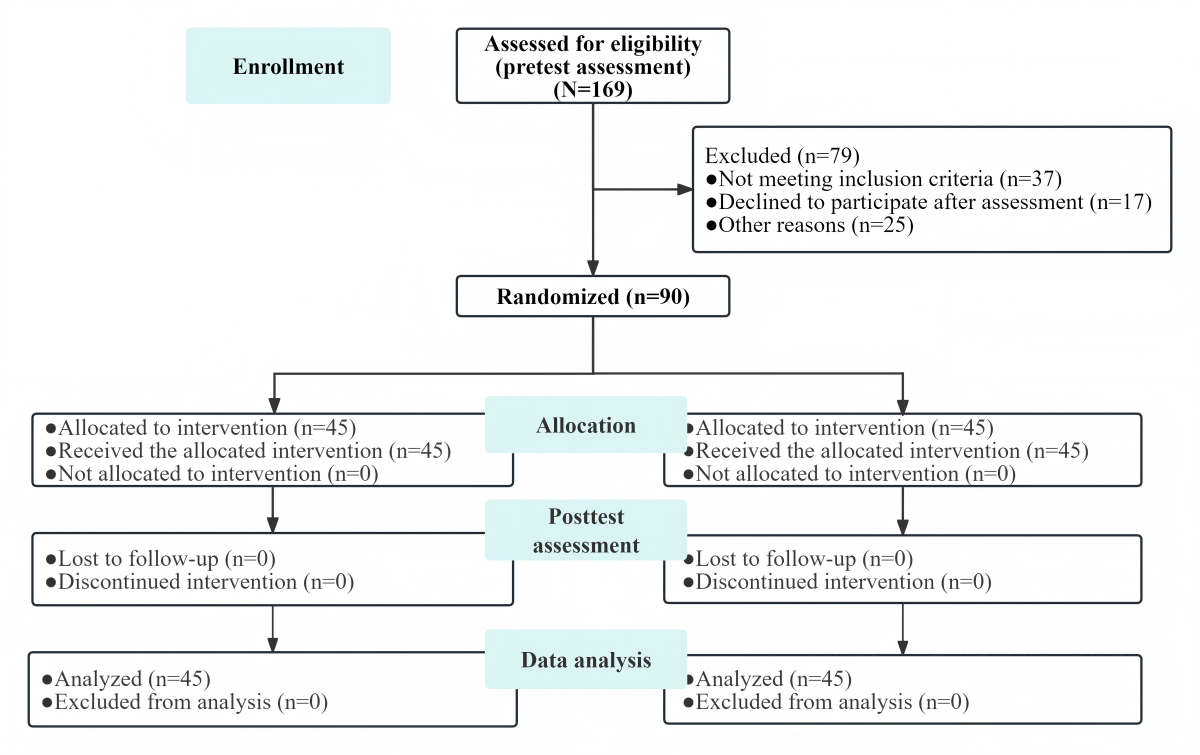
CONSORT (Consolidated Standards of Reporting Trials) flowchart of the study.

The demographic characteristics of the 2 groups of participants are presented in Table 1. A total of 90 participants were included, with 45 (50%) individuals allocated to the intervention and control groups each (Figure 4). This study primarily included female nurses, with 96% (43/45) of them in the control group and 93% (42/45) in the intervention group. The average age was 27.53 (SD 4.0) years in the intervention group and 28.78 (SD 4.8) years in the control group. The demographic characteristics, including sex, age, highest degree, professional title, and work experience, were comparable between the 2 groups. All participants completed both baseline and postintervention assessments.

**Table 1:** Demographic data of the 2 groups (N=90).

Variable			Control group (n=45)		Intervention group (n=45)		*t* test (*df*)		*χ*^*2*^ (*df*)		*P* value
**Sex** **, n (%)**							—^b^		0.2 (1)		.65
	Male	2 (4)		3 (7)							
	Female	43 (96)		42 (93)							
Age (y), mean (SD)^a^			28.78 (4.8)		27.53 (4.0)		1.244 (88)				.19
**Clinical experience** **(** **y), n (%)**							—		4.4 (2)		.11
	≤5	20 (44)		29 (64)							
	6-10	19 (42)		14 (31)							
	≥11	6 (13)		2 (4)							
**Highest degree, n (%)**							—		6.5 (2)		.09
	Diploma or associate degree	6 (13)		13 (29)							
	Bachelor’s degree	38 (84)		30 (67)							
	Master’s degree	1 (2)		2 (4)							
**Professional title, n (%)**							—		0.8 (1)		.37
	Primary	28 (62)		32 (71)							
	Intermediate	17 (38)		13 (29)							
**Work experience in infectious** **disease** **departments, n (%)**							—		1.6 (1)		.21
	Yes	13 (29)		8 (18)							
	No	32 (71)		37 (82)							
**Experience in caring for patients** **with COVID-19 and SARS-CoV-2 infection, n (%)**							—		0.2 (1)		.63
	Yes	34 (76)		32 (71)							
	No	11 (24)		13 (29)							
**Hospital department of work, n (%)**							—		5.5 (3)		.24
	Internal medicine ward	17 (38)		17 (38)							
	Surgical ward	21 (47)		20 (44)							
	Emergency and critical care unit	1 (2)		4 (9)							
	Pediatric department	5 (11)		1 (2)							
	Others	1 (2)		3 (7)							

^a^Age is a continuous variable, expressed as mean (SD).

^b^Not applicable.

### Primary Outcome 1: Comparison of the Theoretical Test Scores Between Groups Before and After Intervention

Before the intervention, no significant difference was found in the score of theoretical tests between the 2 groups (t_88_=1.15*, P*=.25*,* Cohen *d=*0.24; Table 2), using a 2-tailed *t* test. However, after the intervention, the total scores of the theoretical tests in both groups significantly improved compared to the scores before the intervention. However, the 2 groups had no statistically significant difference in scores (t_88_*=*–0.30*, P*=.75, Cohen *d=*–0.06; Table 2).

**Table 2:** The theoretical test scores between groups before and after intervention (N=90).

	Control group (n=45), mean (SD)	Intervention group (n=45), mean (SD)	*t* test (*df*)	*P* value	Cohen *d*
Preintervention score	49.49 (11.46)	46.47 (13.35)	1.15 (88)	.25	0.24^a^
Postintervention score	85.80 (6.49)	86.24 (7.49)	–0.30 (88)	.75	–0.06^b^

^a^According to Cohen conventions, a small effect size of 0.24 indicates a minor difference between the control and intervention groups before the intervention.

^b^Postintervention score Cohen *d* of –0.06: this effect size is negligible, indicating very little or no practical difference between the groups after the intervention.

### Primary Outcome 2 and Secondary Outcomes: Comparison of the Drill Scores and Completion Times Between Groups After Intervention

After the intervention, no statistically significant differences were observed in the drill scores between the 2 groups (all *z* scores=0.00*, P>*.99; Table 3). However, the intervention group demonstrated a notably shorter total duration in completing the 6 target tasks than the control group (t_88_=5.10*,* *P<*.001, Cohen *d*=1.08; Table 3). Among the 6 work tasks, the intervention group completed task 3 (patient reception inwards) and task 6 (leaving the isolation area) significantly faster than the control group (t_88_=3.22*, P=*.002, Cohen *d*=0.68; t_88_=3.03*, P*=.003, Cohen *d*=0.64; Table 3). In comparison, there were no differences in completion times for the remaining 4 tasks between both groups (*P*>.05; Table 3). The *t* test was 2-tailed.

**Table 3:** The drill scores and completion times between groups after intervention (N=90).

Variable		Control group (n=45)	Intervention group (n=45)	*t* test (df)	*z* score	*P* value	Cohen *d*
**Task 1: the nurse enters the isolation ward and reaches the clean area in the nurses’station**							
	Scores, median (IQR)^a^	10 (10-10)	10 (10-10)	—^b^	0.00	>.99	—
	Time taken (min), mean (SD)	3.88 (0.92)	3.62 (0.94)	1.36 (88)	—	.18	0.28^c^
**Task 2: the nurse enters the potentially contaminated area and prepares the necessary supplies**							
	Scores, median (IQR)^a^	20 (18-20)	20 (18-20)	—	0.00	>.99	—
	Time taken (min), mean (SD)	3.79 (0.79)	3.59 (0.80)	1.19 (88)	—	.24	0.25^c^
**Task 3: the nurse enters the patient’s room and completes the reception of the newly admitted patient**							
	Scores, median (IQR)^a^	20 (17.5-20)	20 (18-20)	—	0.00	>.99	—
	Time taken (min), mean (SD)	11.71 (1.71)	10.76 (1.00)	3.22 (88)	—	.002	0.68^d^
**Task 4: the nurse uses the transfer window to deliver items**							
	Scores, median (IQR)^a^	15 (13.5-15)	15 (13.5-15)	—	0.00	>.99	—
	Time taken (min), mean (SD)	2.59 (0.82)	2.48 (0.60)	0.76 (88)	—	.50	0.15^e^
**Task 5: the nurse completes the patient discharge procedures and performs terminal disinfection of the ward after the discharge**							
	Scores, median (IQR)^a^	20 (17-20)	20 (18-20)	—	0.00	>.99	—
	Time taken (min), mean (SD)	9.41 (1.13)	9.24 (1.58)	0.60 (88)	—	.55	0.12^e^
**Task 6: the nurse exits the isolation ward from the contaminated area**							
	Scores, median (IQR)^a^	15 (15-15)	15 (10-15)	—	0.00	>.99	—
	Time taken (min), mean (SD)	7.00 (2.32)	5.59 (2.09)	3.03 (88)	—	.003	0.64^d^
**Total**							
	Scores, median (IQR)^a^	93 (85-98)	92 (88-97)	—	0.00	>.99	—
	Time taken (min), mean (SD)	43.91 (2.99)	40.77 (2.85)	5.10 (88)	—	<.001	1.08^f^

^a^Nonnormally distributed data are presented as median (IQR), using *z* score (Mann-Whitney *U* test).

^b^Not applicable.

^c^Task 1 and task 2: the effect sizes are small (Cohen *d*=0.28 and Cohen *d*=0.25, respectively), indicating minimal to small practical differences in time taken between the 2 groups for these tasks.

^d^Task 3 and task 6: the effect sizes are moderate (Cohen *d*=0.68 and Cohen *d*=0.64, respectively), suggesting that these tasks saw a noticeable improvement in efficiency for the intervention group compared to the control group.

^e^Task 4 and task 5: the effect sizes are very small (Cohen *d*=0.15 and Cohen *d*=0.12, respectively), suggesting very little difference in performance between the groups.

^f^The effect size for the total time taken is large (Cohen *d*=1.08), indicating a significant reduction in overall time taken for completing all tasks by the intervention group, reflecting a substantial practical significance.

## Discussion

### Principal Findings

In this study, we developed an NIVR simulation training program for isolated ward settings aimed at training nurses and assessing the program’s effectiveness through a randomized controlled trial. A total of 90 nurses participated in the training program and underwent pre- and posttraining assessments. The results showed no significant differences between the 2 groups regarding scores of theoretical knowledge and drills after the training (t_88_=–0.30, *P*=.75, Cohen *d*=–0.06; Table 2 and *z* score=0.00, *P>*.99; Table 3). However, the intervention group that received NIVR simulation training significantly reduced their total task completion time compared to the control group (t_88_=5.10, *P<*.001, Cohen *d*=1.08; Table 3). It should be noted that this advantage was only evident in task 3 (receiving patients in the ward: t_88_=3.22, *P*=.002, Cohen *d*=0.68) and task 6 (leaving the isolation ward: t_88_=3.03, *P*=.003, Cohen *d*=0.64; Table 3), with no significant differences in the remaining 4 tasks (all *P*>.05), and the average total time difference was approximately 3 minutes (control group: mean 43.91, SD 2.99 min; intervention group: mean 40.77, SD 2.85 min). These findings suggest that NIVR training is comparable to traditional training in enhancing knowledge and skills and can reduce the time nurses take to complete tasks to some extent. Although the study did not conclusively demonstrate a significant advantage of NIVR training, the findings indicate that VR training can facilitate the effective application of knowledge and skills in real-world settings [34,42]. Moreover, it allows nurses to familiarize themselves with ward environments before pandemics, thus improving their response capabilities. However, further studies are required to confirm other potential benefits. Therefore, it is essential to explore the implementation of VR training to better prepare nurses for future pandemics.

VR training has introduced a highly efficient new method for training nurses in isolation wards. Although traditional training methods, such as using simulated laboratories or observing natural ward settings, are effective, they face challenges including labor intensiveness, financial constraints, resource limitations, time constraints, and risks of cross-infection [13]. Several studies [43,49,51-53] have indicated that VR simulations demonstrate significant potential in pandemic management, covering areas such as infection prevention and control, simulated transmission behaviors, clinical skill training, mental health improvement, and emergency response. These studies have highlighted the vital role of VR technology in managing infectious disease outbreaks such as COVID-19 and Ebola virus disease, leading to enhanced health care professionals’ skills, reduced stress, and improved patient experiences. Our isolation ward VR simulation training program focuses on training nurses to enhance their adaptation to isolation ward settings within a simulated environment, improving their preparedness and capabilities in managing infectious diseases. The training integrates a 2D ward map with dynamic interactions in 3D simulated ward environments through NIVR technology, reconstructing realistic isolation ward scenarios. This innovative training method allows nurses to operate in a safe, noncontact virtual environment, significantly reducing the risk of cross infection [13,25]. This approach aids nurses in comprehensively understanding the unique layout and standardized protocols of isolation wards, significantly enhancing training safety and effectiveness [49,51]. Customized course design helps nurses familiarize themselves with different working environments before entering the wards, alleviating their stress and anxiety [32]. Meanwhile, this highly interactive training allows nurses to realistically simulate various emergencies, strengthening their ability and resilience in handling unexpected events [29]. Importantly, NIVR simulation training supports repetitive practice to improve proficiency in critical skills [26]. Therefore, there is a need to focus on and promote VR technology to comprehensively improve health care workers’ emergency response capabilities and infection control skills.

During outbreaks of infectious diseases, VR training effectively mitigates the challenges posed by contact restrictions encountered in traditional on-site instruction.

In the early stages of the COVID-19 pandemic, restrictions on clinical internships resulted in the widespread adoption of remote virtual simulation technology, particularly in programs where securing face-to-face clinical internships is difficult [39,54-56]. Research by Zhang et al [13] indicates that web-based VR not only meets the training requirements of isolation wards but also effectively enhances nurses’ learning motivation and professional safety [13]. NIVR training, due to its low cost and minimal resource requirements, facilitates remote VR and emerges as an ideal choice for postpandemic nursing education [39]. Our isolation ward virtual simulation training provides an effective learning experience by simulating real environments for learners. The study results show that VR training can replace emergency on-site training, assisting nurses in quickly adapting to their work environment [36,42,57]. Particularly under the circumstances of restricted contact time, remote VR training offers nurses a learning platform unrestricted by time, location, and resources [58,59], improving learning efficiency while enhancing confidence and competence in high-risk areas [42]. Looking ahead, with continuous technological advancements and expanded application scenarios, VR training is poised to play a more significant role in nursing education and medical training. It has the potential to enhance health care professionals’ skills and provide more practice opportunities while simultaneously ensuring greater safety for both medical personnel and patients.

Studies indicate that interactive VR training can enhance health care professionals’ clinical decision-making skills and critical thinking, thereby improving efficiency and quality of care [27,29,30,60]. In high-risk, high-workload isolation wards, effective time management and operational efficiency are particularly crucial for nurses. Our study shows that nurses who underwent VR training required less time to complete specific tasks, such as entering the wards to receive patients and exiting isolation zones (t_88_=3.22, *P*=.002, Cohen *d*=0.68; t_88_=3.03, *P*=.003, Cohen *d*=0.64, respectively; Table 3). This suggests that VR training may offer advantages in managing complex tasks. However, there are still debates regarding the effectiveness of VR. While some studies highlight VR’s ability to effectively enhance nursing students’ knowledge levels, it has not yet significantly outperformed traditional methods in skills, satisfaction, confidence, and performance time [38,42,61]. Therefore, policy makers should proceed cautiously when promoting VR training and consider integrating various training methods to ensure comprehensive skill improvement for nurses. Future research can further validate the effectiveness of VR training through large-scale, multicenter, randomized controlled trials and explore its application advantages and limitations in specific medical contexts. In addition, establishing task efficiency assessment mechanisms and delving into the long-term effects and cross-disciplinary applications of VR training can further elevate training outcomes.

### Cost-Benefit Considerations

In this study, although both groups had the same training duration, the cost of developing VR training programs is an important factor that needs to be considered. While NIVR training significantly enhanced caregivers’ coping skills, its initial development costs were relatively higher compared to traditional training methods. However, as the number of trainees increases, these initial investments are progressively amortized, reducing the average cost per trainee. Farra et al [62] noted that despite the average cost per person for VR training being US $327.78 in the first year, which was US $97.99 higher than the US $229.79 for live drills, the cost gradually decreased in the subsequent years. After 3 years, the average cost of VR training had dropped to US $115.43 per person, while live drills remained at US $229.79, resulting in savings of approximately US $114.36 per person for VR training. Due to its reusability, VR demonstrated greater cost-effectiveness over a three-year period, proving to be more economically advantageous in the long run compared to traditional on-site practice [62,63]. Halfer and Rosenheck [64] further supported this view by discovering that although the development cost of a VR program was higher than that of paper floor plans, it significantly enhanced nurses’ efficiency in navigating a new hospital building and reduced the required practice time. Specifically, the total cost for traditional methods was US $570,000, whereas it was US $421,000 for the virtual methods. Therefore, researchers and decision makers need to carefully weigh the cost and impact of VR training, considering its long-term feasibility and effectiveness in medical training. Future cost-benefit analyses will provide crucial support for these evaluations. A comprehensive assessment of VR education’s economic efficiency and academic value is essential for determining its long-term viability when considering implementation costs and training outcomes.

### Limitations

This study has several limitations. First, the small sample size limits the generalizability of the results, indicating a need for future research with larger samples. Second, the training content and methods are preliminary and require further refinement to address diverse nursing scenarios. Third, the absence of a baseline speed measure before training leaves open the possibility that observed group differences may partly arise from factors unrelated to the training method. This should be considered when assessing our findings. Moreover, the lack of blinding for assessors introduces potential bias in performance evaluations. Future studies should use blinded assessments to enhance reliability. Although VR simulation training appears promising, further research with improved measures of work efficiency and controlled conditions is essential to validate and expand these initial findings.

### Conclusions

This study developed an NIVR simulation training program for isolation wards and evaluated its effectiveness through a randomized controlled trial. While the NIVR simulation training demonstrates faster performance in certain tasks, it shows no significant differences in theoretical and drill scores compared to traditional training. Nevertheless, its safety, repeatability, and realistic simulated environment suggest potential for pandemic response training. Despite not significantly outperforming traditional training methods, NIVR simulation training can still facilitate the practical application of knowledge and skills. It helps nurses familiarize themselves with the ward environment in advance, thus improving their response capabilities. Future research should focus on optimizing training programs and validating and enhancing VR training through large-scale, multicenter randomized controlled trials. In addition, establishing mechanisms for task efficiency evaluation and exploring its long-term effects and interdisciplinary applications will enhance pandemic preparedness.

## Data Availability

The datasets generated during and analyzed during this study are not publicly available due to participant privacy concerns but are available from the corresponding author upon reasonable request.

## Appendix

Multimedia Appendix 1 CONSORT-eHEALTH checklist (V 1.6.1).

## Abbreviations

NIVR: nonimmersive virtual reality
OSCE: Objective Structured Clinical Examination
VR: virtual reality
